# Restoring MLL reactivates latent tumor suppression-mediated vulnerability to proteasome inhibitors

**DOI:** 10.1038/s41388-020-01408-7

**Published:** 2020-07-30

**Authors:** Maolin Ge, Dan Li, Zhi Qiao, Yan Sun, Ting Kang, Shouhai Zhu, Shifen Wang, Hua Xiao, Chunjun Zhao, Shuhong Shen, Zhenshu Xu, Han Liu

**Affiliations:** 1 Shanghai Institute of Hematology, State Key Laboratory of Medical Genomics, National Research Center for Translational Medicine at Shanghai, Ruijin Hospital affiliated to Shanghai Jiao Tong University School of Medicine, 200025 Shanghai, China; 2 State Key Laboratory of Microbial Metabolism, School of Life Sciences and Biotechnology, Shanghai Jiao Tong University, 200240 Shanghai, China; 3 Department of Oncology, Xin Hua Hospital, School of Medicine, Shanghai Jiao Tong University, 200092 Shanghai, China; 4 Fujian Institute of Hematology, Fujian Provincial Key Laboratory of Hematology, Fujian Medical University Union Hospital, 350001 Fuzhou, China; 5 Key Laboratory of Pediatric Hematology and Oncology Ministry of Health, Department of Hematology & Oncology, Pediatric Translational Medicine Institute, Shanghai Children’s Medical Center, School of Medicine, Shanghai Jiao Tong University, 200127 Shanghai, China

**Keywords:** Cancer therapeutic resistance, Acute lymphocytic leukaemia

## Abstract

MLL undergoes multiple distinct chromosomal translocations to yield aggressive leukemia with dismal outcomes. Besides their well-established role in leukemogenesis, MLL fusions also possess latent tumor-suppressive activity, which can be exploited as effective cancer treatment strategies using pharmacological means such as proteasome inhibitors (PIs). Here, using MLL-rearranged xenografts and MLL leukemic cells as models, we show that wild-type MLL is indispensable for the latent tumor-suppressive activity of MLL fusions. MLL dysfunction, shown as loss of the chromatin accumulation and subsequent degradation of MLL, compromises the latent tumor suppression of MLL-AF4 and is instrumental for the acquired PI resistance. Mechanistically, MLL dysfunction is caused by chronic PI treatment-induced epigenetic reprogramming through the H2Bub-ASH2L-MLL axis and can be specifically restored by histone deacetylase (HDAC) inhibitors, which induce histone acetylation and recruits MLL on chromatin to promote cell cycle gene expression. Our findings not only demonstrate the mechanism underlying the inevitable acquisition of PI resistance in MLL leukemic cells, but also illustrate that preventing the emergence of PI-resistant cells constitutes a novel rationale for combination therapy with PIs and HDAC inhibitors in MLL leukemias.

## Introduction

The mixed-lineage leukemia (*MLL*) gene encodes a histone methyltransferase governing histone H3 lysine residue 4 (H3K4) methylation [1]. MLL orchestrates several essential cellular processes by positively regulating its target genes, especially the *Hox* gene family and cell cycle genes [2, 3]. MLL precursor polypeptide is site-specifically cleaved by the Taspase1 protease and functions as heterodimeric complexes composed of its amino (MLL^N320^) and carboxy (MLL^C180^) terminal subunits [4, 5]. The *MLL* gene undergoes many distinct chromosomal rearrangements to yield aggressive acute lymphoblastic leukemia (ALL) and acute myeloid leukemia (AML). Leukemogenic MLL translocations fuse the N-terminal l~1400 amino acids of MLL in frame with more than 94 translocation partner genes, which are present at high frequency in infants and at lower frequencies in children and adults [5, 6].

In contrast to the rearranged allele, the other *MLL* allele usually remains intact and expressed. The contribution of this wild-type MLL allele to leukemogenesis in MLL-rearranged leukemias has been the subject of intense research. Several lines of investigation support that endogenous MLL maintains the H3K4me status and facilitates MLL-fusion protein-mediated leukemogenesis [7–9]. Meanwhile, the loss of endogenous MLL alone can have significant impacts on several AML subtypes, including those initiated by MN1 and NUP98 fusion proteins [10, 11]. However, other studies have demonstrated that endogenous MLL is dispensable for MLL-rearranged AML and that MLL deletion alone had no major impact on the survival of MLL leukemic cells [12, 13]. Nevertheless, these discrepancies occur mainly in AML models, while the contribution of the wild-type allele of MLL to MLL-rearranged ALL remains elusive.

The improved molecular understanding of MLL and MLL fusions has led to the identification of several potential mechanism-based therapeutic targets. While the necessity of the wild-type allele of MLL for leukemogenesis is debatable, it has nonetheless become an attractive therapeutic target in MLL leukemia. Given the findings that the remaining wild-type MLL protein is generally much less abundant than the MLL fusions in MLL leukemia cells, several candidate therapeutic strategies are emerging that stabilize wild-type MLL protein to displace MLL chimeras from chromatin and therefore evade the oncogenic addiction of these cells to MLL chimeras [14, 15]. For example, the inhibition of interleukin-1 receptor-associated kinases (IRAKs) impedes UBE2O-mediated MLL degradation and stabilizes wild-type MLL protein. Casein kinase II (CKII) inhibition, on the other hand, blocks the phosphorylation of the taspase1 cleavage site on MLL and inhibits taspase1-dependent MLL processing, thus increasing MLL stability. Analogously, IRAK and CKII inhibition induce wild-type MLL to outcompete the oncogenic MLL chimeras through additional chromatin-binding modules, such as PHD fingers and a bromodomain. These domains are not retained in MLL fusions but exist exclusively in wild-type MLL [16]. Histone deacetylase (HDAC) inhibitors have also been reported to activate wild-type MLL [17], but the underlying mechanisms are not fully understood.

Proteasome inhibitors (PIs) are newly reported clinical regimens for MLL therapy, specifically MLL-r B-ALL cells, but not AML [18, 19]. Mechanistically, proteasome inhibition induces the intrinsic tumor-suppressive activity of MLL fusions by triggering apoptosis and cell cycle arrest involving cleavage of BID by caspase-8 and upregulation of p27, respectively [18, 20]. The accumulation of endogenous MLL-fusion proteins at the p27 locus through PAX5 is decisive to the specific cytotoxicity caused by proteasome inhibition in lymphoid, but not myeloid, MLL leukemias.

We previously reported that PI bortezomib single-agent therapy showed effectiveness in mouse models and patients with pro-B MLL leukemia; however, the inevitable emergence of PI resistance imposes limits on bortezomib’s clinical application [18]. Therefore, identification of the mechanism underlying PI resistance and the design of novel combination strategies are essential to overcome resistance and facilitate the application of PIs to MLL leukemias. Intriguingly, we found that the wild-type MLL protein was less abundant and was insensitive to PI treatment in resistant MLL leukemia cells, compromised the latent tumor suppression of MLL fusions. Therefore, we reasoned that disruption of the balance between wild-type MLL and MLL chimeras plays a critical role in PI resistance, and that targeting MLL dysfunction and restoring MLL may be a promising strategy for treating the aggressive resistance in MLL leukemia.

## Results

### PI resistance is associated with the failure of MLL and p27 induction

**Fig. 1:**
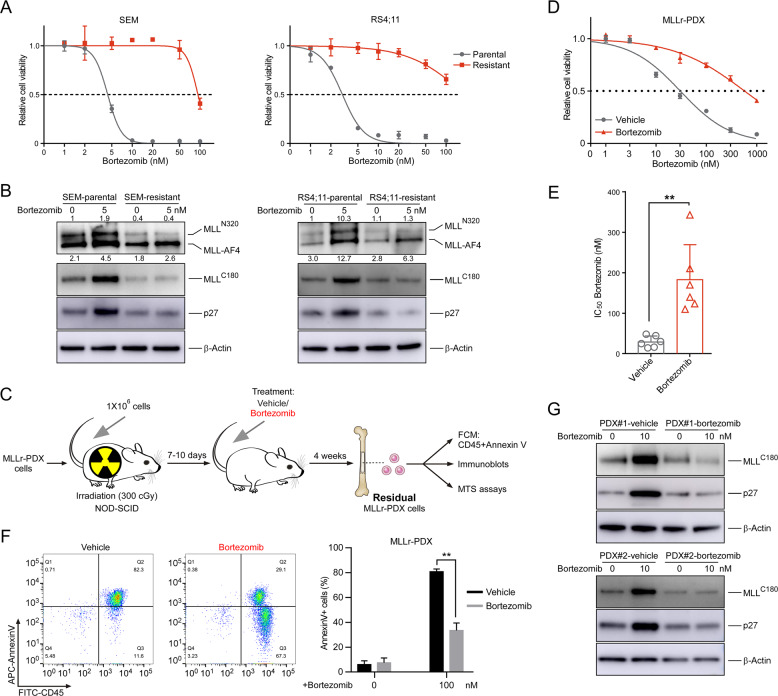
PI resistance is associated with the failure of MLL and p27 induction. **a** Cell viability of parental and resistant SEM and RS4;11 cells was measured with MTS assay 24 h after the addition of bortezomib at the indicated concentrations. **b** Immunoblots of the indicated cells before and after a 12 h exposure to bortezomib. The band intensity represents fold changes to internal control by using Image J software analysis. The results are shown as fold induction of MLL^N320^ and MLL-AF4 relative to endogenous wild-type MLL control. **c** Schematic for the different treatment strategies on the MLL-r PDX cells. **d**, **e** Cell viability of bone marrow cells derived from MLL-r PDX mice was measured with MTS assay 24 h after the addition of bortezomib. One representative of six independent experiments is shown in (**d**). Histogram of six independent biological replicates with three technical replicates is shown in (**e**). **f** Bone marrow cells from the MLL-r PDX mice were obtained and dead cells were removed. Flow cytometric detection of CD45 expression and apoptosis was measured 16 h after the addition of bortezomib. **g** Immunoblots of the indicated PDX cells before and after a 12 h exposure to the indicated concentrations of bortezomib. **P < 0.01; two-tailed *t*-test. Data are represented as mean ± SD.

We previously observed that pro-B MLL leukemia displayed selective sensitivity to the PI bortezomib, but the disease did eventually develop in xenograft mice [18]. To understand mechanisms by which pro-B MLL leukemic cells overcome proteasome inhibition, we modeled bortezomib-resistant SEM and RS4;11 cells by continuously treated parental cells with a sublethal dose of bortezomib (5 nM) for over 4 weeks (Figs. 1a and S1A). Cells were passaged and the inhibitor was replenished every 3 days. The half-maximal dosage effect (IC_50_) values were measured intermittently until the drug resistance was acquired. In PI-sensitive MLL leukemic cells, bortezomib induces the accumulation of MLL-AF4 and latent tumor suppression programs, triggering cell cycle arrest involving the activation of the p27 gene (*CDKN1B*) [18]. Like the original PI-sensitive parental cells, the abundance of MLL-AF4 in resistant cells exhibited a remarkable increase under bortezomib treatment, while the abundance of wild-type MLL and p27 did not increase significantly (Fig. 1b), indicating that PI resistance in MLL leukemic cells is associated with the failure of MLL and p27 induction. Interestingly, while the activation of p27 is essential for cell cycle deregulation caused by acute PI treatment, it was not significantly activated in chronically induced PI-resistant cells (Fig. 1b), suggesting that the cell cycle deregulation in PI-resistant cells was achieved through an alternative mechanism.

To determine if the failure of MLL and p27 induction was present in patient-derived cells, we generated MLL-rearranged patient-derived xenografts (MLL-r PDX) from two MLL-fusioned B-ALL patients (Fig. S1B, C, Table S1). We collected the bone marrow cells from xenograft mice treated with bortezomib and evaluated the sensitivity of these cells to bortezomib (Fig. 1c). Compared with the xenograft mice treated with vehicle, cell viability of residual MLL-r PDX cells surviving bortezomib exposure was notably increased (Fig. 1d, e). We then examined the apoptosis of these residual cells, and the results showed that bortezomib treatment significantly reduced the apoptosis of bortezomib-treated MLL-r PDX cells, indicating that the residual MLL-r PDX cells surviving bortezomib exposure showed resistance to bortezomib (Fig. 1f). We further examined the expression of wild-type MLL and p27 proteins. Compared with the xenograft mice treated with vehicle, the induction of wild-type MLL and p27 was not significant in the bortezomib-treated MLL-r PDX mice (Fig. 1g). Hence, these results indicate that PI resistance is associated with the failure of MLL and p27 to be activated and cause cell cycle arrest and subsequent apoptosis.

**Fig. 2:**
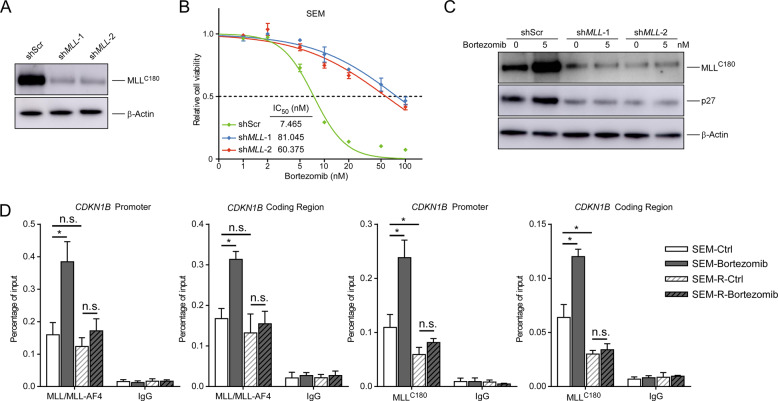
MLL reduction leads to p27 down-regulation and PI resistance. **a** The MLL protein levels of SEM cells infected by the indicated lentiviral vectors. **b** Cell viability of SEM cells infected by the indicated lentiviral vectors. The IC_50_ of different cells was quantified. **c** Immunoblots of SEM cells infected by the indicated lentiviral vectors before and after a 12 h exposure to bortezomib. **d** ChIP analyses at the promoter and coding region of the *CDKN1B* locus in the indicated cells upon bortezomib treatment. MLL^N320^ (D2M7U) antibody against the shared amino terminus of MLL and MLL-AF4 was utilized. R resistant; n.s. not significant; *P < 0.05; two-tailed *t*-test. Data are represented as mean ± SD.

The transcriptional regulation of p27 by MLL and MLL fusions has been reported in various experimental settings [18, 21, 22]. To confirm the association of MLL suppression with PI sensitivity and p27 expression in MLL leukemia, we knocked down the *MLL* gene in SEM cells and observed that MLL depletion significantly decreased the sensitivity of SEM cells to bortezomib (Fig. 2a, b), reduced the expression of p27 and suppressed the induction of p27 by bortezomib (Fig. 2c). We further performed chromatin immunoprecipitation (ChIP) assays to examine the occupancy of MLL and MLL/MLL-AF4 at both the *CDKN1B* promoter and coding regions. In fact, in bortezomib-treated SEM cells, the binding of MLL/MLL-AF4 and MLL at both the *CDKN1B* promoter and coding region was increased (Fig. 2d). However, bortezomib-induced accumulation of MLL/MLL-AF4 and MLL was abolished in PI-resistant cells (Fig. 2d). Moreover, compared to parental cells, PI-resistant cells showed a significantly decreased level of *CDKN1B* promoter- and coding region-bound MLL, but not that of MLL/MLL-AF4 (Fig. 2d). Taken together, our data favor a positive correlation between MLL and p27 expression, indicating that MLL dysfunction compromises the latent upregulation of p27 by MLL-AF4 and is conducive to acquire PI resistance.

### The cell cycle of PI-resistant MLL leukemic cells is dysregulated

**Fig. 3:**
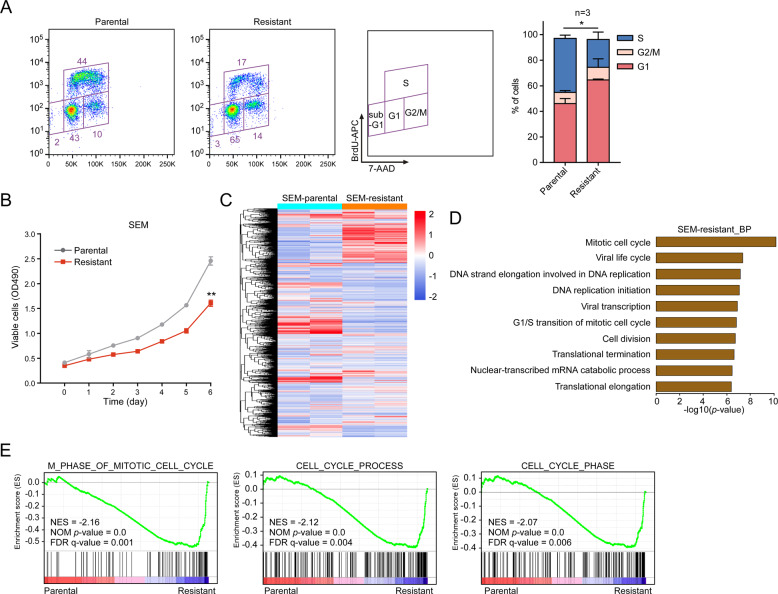
The cell cycle of PI-resistant MLL leukemic cells is dysregulated. **a** Cell cycle profiling of SEM parental and resistant cells. Stacked barplot shows the fraction of cells viable in G1, S and G2/M phases of the indicated cells (three independent biological replicates with three technical replicates each). **b** The proliferation of SEM parental and resistant cells for 6 days. **c** Unsupervised hierarchical clustering heatmap of differentially expressed genes (DEGs) in SEM parental and resistant cells. **d** Biological process (BP) enrichment analysis of DEG datasets obtained from SEM parental and resistant cells. GO analysis was performed with DAVID, and items were ordered by *P* value. **e** The DEGs of SEM parental and resistant cells were studied with GSEA analysis. Normalized enrichment score (NES), nominal (NOM) *P* value, and false discovery rate (FDR) are indicated. **P < 0.01; two-tailed *t*-test. Data represent the means of triplicate reactions ± SD.

MLL is involved in the positive regulation of cell cycle control [2, 3]. To understand the effects of MLL dysfunction on cell cycle progression of PI-resistant cells, we examined the cell cycle and proliferation. As expected, the PI-resistant MLL leukemic cells SEM and RS4;11 were slow cycling with a low S-phase fraction (Figs. 3a and S2A), and cell proliferation was significantly suppressed (Figs. 3b and S2B). We subsequently performed RNA sequencing (RNA-Seq) to further characterize these PI-resistant cells (Figs. 3c and S2C). Upon functional annotation clustering using DAVID, enrichment of genes involved in the mitotic cell cycle, G1/S transition, and negative regulation of cell proliferation was observed (Figs. 3d and S2D). Using gene set enrichment analysis (GSEA), we established that genes associated with cell cycle processes were significantly enriched in both SEM and RS4;11 PI-resistant cells (Figs. 3e and S2E, Tables S2 and S3). Taken together, these results indicate that the cell cycle progression of PI-resistant cells mediated by MLL dysfunction is notably blocked.

### PI induces MLL dysfunction through epigenetic reprogramming

**Fig. 4:**
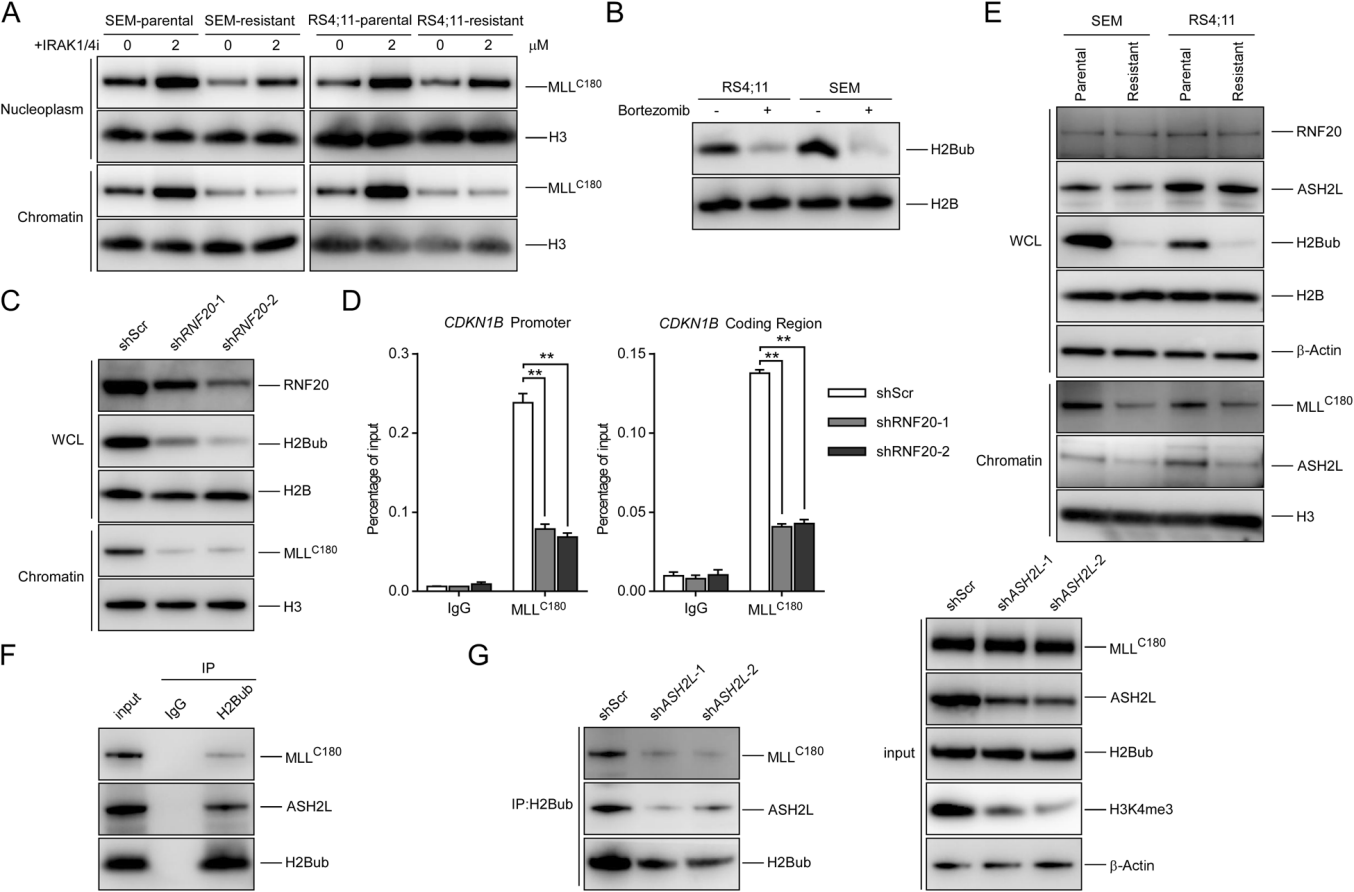
PI induces MLL dysfunction through H2Bub depletion. **a** The indicated cells were treated with IRAK1/4 inhibitor for 48 h. Nucleoplasm and chromatin-bound fractions were purified and immunoblots of the indicated antibodies were analyzed. **b** Immunoblots of H2Bub treated with bortezomib (10 nM) for 8 h in the indicated cells. **c** SEM cells were infected by the indicated lentiviral vectors. ChIP analyses at the promoter and coding region of the *CDKN1B* locus in the indicated cells were detected. **d** SEM cells were infected by the indicated lentiviral vectors. Immunoblots of WCL and chromatin-bound proteins were detected. **e** Immunoblots of whole-cell lysates (WCL) and chromatin-bound proteins in the indicated SEM or RS4;11 cells. **f** SEM cells subjected to anti-H2Bub immunoprecipitation (IP) and analyzed with the indicated antibodies. **g** SEM cells of the indicated knockdown were subjected to anti-H2Bub IP and analyzed with the indicated antibodies.

Since *MLL* mRNA was not significantly decreased (Fig. S3A) and MLL protein was less stable in PI-resistant cells (Fig. S3B), we examined whether PI resistance could be reversed by restoring the protein level of MLL using the IRAK1/4 inhibitor [14, 23]. Surprisingly, even though MLL protein was notably induced by IRAK1/4 inhibitor in PI-resistant cells (Fig. S3C), the sensitivity to bortezomib could not be rescued (Fig. S3D). Interestingly, upon IRAK1/4 inhibitor treatment, while both chromatin-bound and free nucleoplasmic MLL were proportionally increased in the parental cells, only nucleoplasmic MLL was increased in the resistant cells (Fig. 4a). Consistent with this, less MLL was chromatin-bound in the resistant cells (Fig. 4a). Since stabilized MLL could accumulate on chromatin to trimethylate H3K4 [3, 14], our results that stabilized MLL failed to accumulate on chromatin suggested unfavorable chromatin accessibility for MLL in these resistant cells, which suppresses the effect of IRAK1/4 inhibitor on PI-resistant cells. Furthermore, chromatin-associated MLL was more stable than the nucleoplasmic MLL (Fig. S3E). Taken together, these results suggest that in PI-resistant cells, it is the unfavorable chromatin accumulation of MLL that caused less MLL to be recruited onto chromatin, resulting in the degradation and reduction of total MLL protein, but not vice versa.

We next investigated how the chromatin accumulation of MLL was reduced upon PI treatment. Consistent with previous reports, we observed that PI treatment depleted ubiquitinated histone H2B (H2Bub) [24] in RS4;11 and SEM cells (Fig. 4b). RNF20 is the major E3 ubiquitin ligase for histone H2B and RNF20 depletion causes a global reduction in H2Bub level [25]. We further examined whether the reduction in H2Bub caused by depleting RNF20 could impair the recruitment of MLL to chromatin. Indeed, upon RNF20 depletion and a reduction in H2Bub levels, the chromatin-bound MLL and *CDKN1B* locus-bound MLL were significantly decreased (Fig. 4c, d). We further observed that H2Bub in PI-resistant cells was reduced to lower levels than in parental cells, showing a good correlation with the amount of chromatin-bound MLL (Fig. 4e). However, there was no significant change in RNF20 expression, suggesting that the reduction in chromatin-bound MLL is caused by PI treatment-induced depletion of H2Bub, rather than by RNF20 reduction.

Next, we addressed how H2Bub depletion could impair the recruitment of MLL to chromatin. It has been reported that ASH2L in the MLL complex-mediated H2Bub-dependent H3K4 methylation [26–28]. Similar to chromatin-bound MLL, chromatin-bound ASH2L was also decreased in PI-resistant cells (Fig. 4e). Moreover, our co-immunoprecipitation assays confirmed that ASH2L and MLL could be recruited to H2Bub (Fig. 4f). We, therefore, hypothesized that PI treatment may decrease the H2Bub level to prevent ASH2L-mediated recruitment of MLL to chromatin. To confirm the critical role of ASH2L in mediating the recruitment of MLL to H2Bub, we depleted ASH2L and observed that the recruitment of MLL to H2Bub was abolished (Fig. 4g). The results indicate that the depletion of H2Bub induced by PI treatment decreases ASH2L-mediated recruitment of MLL to chromatin and causes the deregulation of a subset of cell cycle-associated genes.

### HDAC inhibitors restore cell sensitivity by enhancing the chromatin accessibility of MLL

Our previous results showed that MLL fusions functioned as dominant-negative mutants by preventing the stabilization of wild-type MLL [3]. While the IRAK1/4 inhibitor can recruit stabilized MLL to chromatin and displace MLL-fusion protein in 293T cells [14], it failed to do so in the resistant cells because of an epigenetic barrier (Fig. 4a). In order to develop an effective epigenetic therapy strategy that can tackle the resistance, we sought an epigenetic drug that could increase chromatin-bound MLL in the resistant cells. MLL possesses several putative chromatin targeting domains including three AT-hooks, one CXXC domain, four PHDs, and one bromodomain [29, 30]. Unlike the AT-hooks and CXXC domain, the PHDs and bromodomain are not retained in MLL fusions and exist exclusively in wild-type MLL [16]. Therefore, we focused on epigenetic changes that could specifically enhance chromatin accessibility to the PHDs and bromodomain, so that it would be clinically translatable to MLL leukemia.

**Fig. 5:**
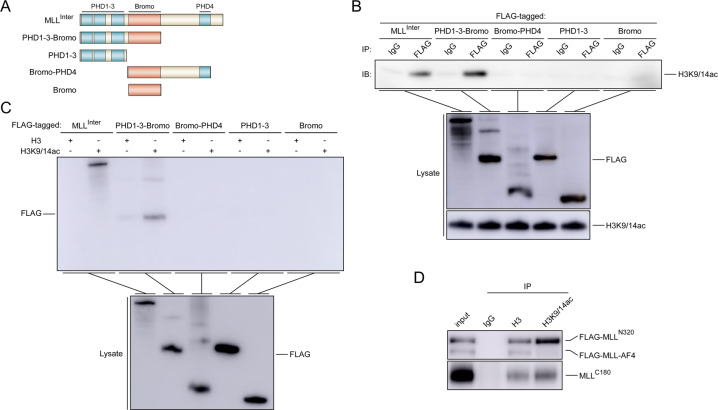
MLL interacts with H3K9/14ac. **a** The construction of MLL domains. 293T cells were transfected with the indicated plasmids for 24 h and subjected to co-immunoprecipitation assays (**b**) or biotin-conjugated histone tail pull-down assays (**c**). The co-precipitated protein was detected by immunoblots. **d** 293T cells co-transfected with the indicated plasmids for 24 h were subjected to co-immunoprecipitation assays. The co-precipitated protein was detected by immunoblots.

It has been noted that wild-type MLL can be activated by class I HDAC inhibitors and displace MLL-AF4 from the promoter [17]. We thus reasoned that histone acetylation might facilitate chromatin accessibility for MLL. Bromodomains are well documented as acetyl-lysine-reading modules [31]. Although previous reports showed that MLL-Bromo was unable to recognize acetylated lysine residues after acetylation of H4K12, H4K16, H3K23, and H3K27 [30], our co-immunoprecipitation assays and peptide pull-down assays demonstrated that PHD1–3 along with Bromo could directly interact with H3K9/14ac (Fig. 5a–c). We further confirmed that wild-type MLL, but not MLL-fusion proteins, interacted with H3K9/14ac (Fig. 5d). In agreement with this, it has been reported that H3K14ac is required for H3K4 trimethylation by COMPASS, an evolutionarily conserved histone methyltransferase complex containing an MLL ortholog [32]. Taking these results together, we hypothesized that the altered chromatin state using HDAC inhibitors in PI-resistant cells might overcome bortezomib resistance.

**Fig. 6:**
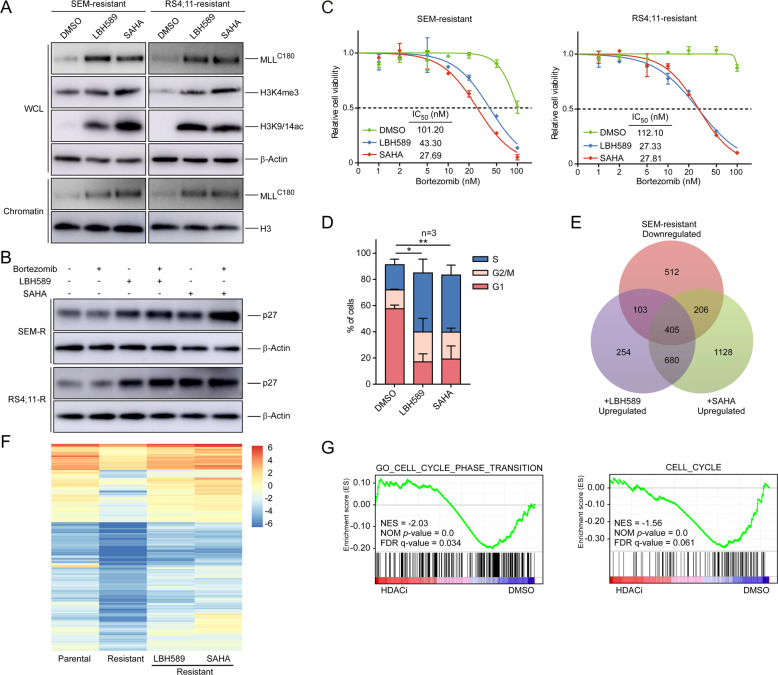
HDAC inhibitors restore cell sensitivity by enhancing the chromatin accumulation of MLL. **a** SEM-resistant and RS4;11-resistant cells were treated with DMSO, the HDAC inhibitor LBH589 (5 nM for SEM and 50 nM for RS4;11), or SAHA (2 μM) for 20 h. Immunoblots of the indicated antibodies were analyzed. **b** SEM-resistant (SEM-R) and RS4;11-resistant (RS4;11-R) cells were treated with HDAC inhibitors described in (**a**), and then treated with 5 nM bortezomib for 12 h. Immunoblots of the indicated antibodies were analyzed. **c** The indicated cells were treated with DMSO or HDAC inhibitors for 20 h and cell viability to bortezomib was measured. **d** Cell cycle profiles of SEM-resistant cells treated with DMSO, LBH589 (5 nM), or SAHA (2 μM) for 20 h. Venn diagram (**e**) and heatmap of the overlapped 405 genes (**f**) of SEM parental and resistant cells treated with DMSO, LBH589 (5 nM), or SAHA (2 μM) for 20 h. **g** GSEA analysis of datasets obtained from SEM-resistant cells after the treatment of DMSO, LBH589 (5 nM), or SAHA (2 μM). *P < 0.05; **P < 0.01; two-tailed *t*-test. Data represent the means of triplicate reactions ± SD.

The HDAC inhibitor agents panobinostat (LBH589) and vorinostat (SAHA) are currently being tested in combination with various anticancer therapies [33–35]. Notably, when PI-resistant leukemic cells were treated with LBH589 or SAHA, we observed an increase in H3K9/14ac, chromatin-associated MLL, and H3K4me3 expression (Fig. 6a). Moreover, HDAC inhibitors significantly induced *CDKN1B* locus-bound H3K9/14ac and MLL, correlated with an increase in p27 activation (Figs. 6b and S4A). In order to determine whether HDAC inhibitor-induced upregulation of p27 is MLL dependent, we knocked down the *MLL* gene in PI-resistant SEM cells (Fig. S4B) and observed that MLL depletion significantly suppressed the induction of p27 by HDAC inhibitors (Fig. S4C). These data suggest that HDAC inhibitors lead to MLL accumulation on chromatin and directly promote the expression of the *CDKN1B* gene and restore p27 induction by PIs in PI-resistant cells.

To investigate the therapeutic potency of HDAC inhibitors in PI-resistant cells, we examined the effect of HDAC inhibitors in combination with bortezomib. The results demonstrated that co-treatment of bortezomib with LBH589 or SAHA led to a strong reduction in cell viability (Fig. 6c), which was correlated with marked induction of cells in S-phase (Figs. 6d and S4D). Total RNA-seq revealed that in the presence of HDAC inhibitors, a large proportion of downregulated genes in SEM and RS4;11-resistant cells were notably induced (Figs. 6e, f and S4E, F, Tables S4 and S5). To determine the set of genes commonly deregulated in PI-resistant cells under HDAC inhibitor treatment, we analyzed the overlap of differentially expressed genes (DEGs) and gene sets. Venn diagram analysis identified 1959 common deregulated genes by both HDAC inhibitors in RS4;11 and SEM-resistant cells (Fig. S5A). Among them, 1323 common upregulated genes and 425 common downregulated genes were overlapped in HDAC inhibitor-treated RS4;11 and SEM-resistant cells (Fig. S5B). Further analysis showed the overlap of DEGs involved in multiple biological processes, of which the cell proliferation pathway was among the top enriched terms (Fig. S5C, D). These results reveal that under HDAC inhibitor treatment, a large number of genes in different MLL-resistant cells were commonly induced, involving in several biological processes such as cell proliferation. Using GSEA, we established that the genes associated with cell cycle processes were significantly enriched in the treatment sets that were upregulated by HDAC inhibitors (Fig. 6g, Table S6), indicating that HDAC inhibitors restored the expression patterns of cell cycle genes. Collectively, these findings raise the possibility that a combinational therapy strategy using PIs and HDAC inhibitors may be effective in treating MLL-resistant cells.

### The combination of PI and HDAC inhibitor overcomes PI resistance in vivo

**Fig. 7:**
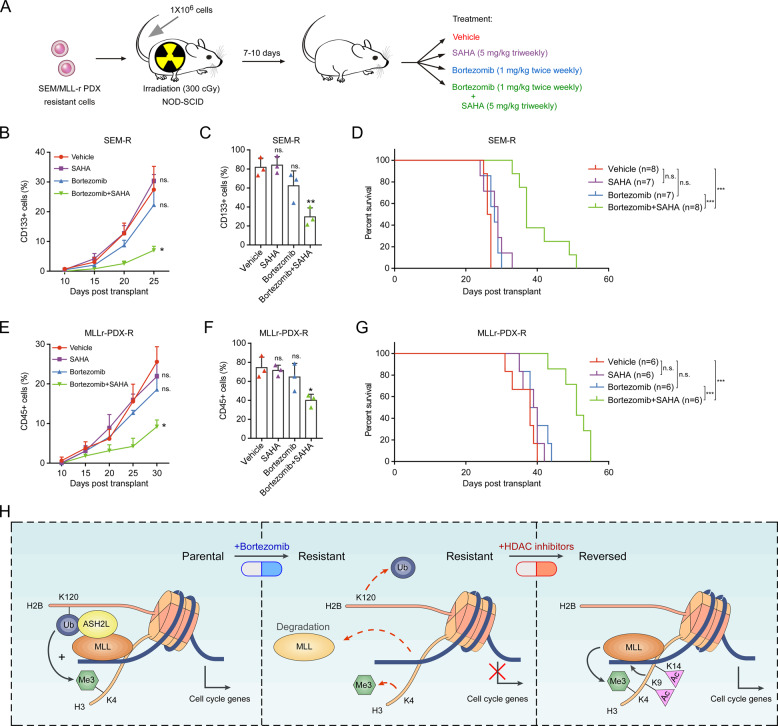
The combination of PI and HDAC inhibitor overcomes PI resistance in vivo. **a** Treatment schedule for administration of bortezomib, SAHA, or vehicle. **b**, **c** Analysis of the recipient mice transplanted with SEM-resistant cells. The percentage of leukemic cells in the peripheral blood (**b**) or bone marrow (**c**) of the indicated groups of mice was monitored using CD133 antibody. The bone marrow cells from xenograft mice were collected 25 days post transplant. **d** Kaplan–Meier survival curves for the SEM resistance model from enrollment time. **e**, **f** Analysis of the recipient mice transplanted with PI-resistant MLL-r PDX cells. The percentage of leukemic cells in the peripheral blood (**e**) or bone marrow (**f**) of the indicated groups of mice was monitored using CD45 antibody. The bone marrow cells from xenograft mice were collected 30 days post transplant. **g** Kaplan–Meier survival curves for the MLL-r PDX resistance model from enrollment time. **h** The illustration depicts how proteasome inhibition induces resistant cells and the therapeutic function of HDAC inhibitors in PI resistance. n.s. not significant; *P < 0.05; **P < 0.01; ***P < 0.001; by two-tailed *t*-test or log-rank test for significance. Data represent the means of triplicate reactions ± SD.

Next, we transplanted the PI-resistant SEM cells into NOD-SCID mice and evaluated the in vivo efficacy of bortezomib alone or combination with SAHA [35] (Fig. 7a). Bortezomib single-agent treatment showed no benefit on these engrafted mice, consistent with the idea that these cells were refractory to bortezomib treatment. In contrast, mice treated with bortezomib in combination with SAHA showed notable responsiveness (Fig. 7b, c) and had a significant improvement in overall survival (Fig. 7d). We further performed the in vivo efficacy detection of combined therapy using MLL-r PDX cells. The monitored peripheral blood and bone marrow results showed that bortezomib in combination with SAHA significantly suppressed the progression of MLL leukemia (Fig. 7e, f). Moreover, compared with bortezomib or SAHA monotherapy, combined therapy significantly increased the overall survival of xenograft mice (Fig. 7g). Furthermore, immunoblot results showed that SAHA monotherapy significantly induced H3K9/14ac, chromatin-bound MLL and H3K4me3 in PI-resistant SEM cells and MLL-r PDX cells (Fig. S6A), while the induction of combined treatment was more obvious, accompanied by significant apoptosis (Fig. S6A, B), indicating that HDAC inhibitors can induce H3K9/14ac and lead to the accumulation of MLL on chromatin to restore PI resistance. These results strongly suggest that a combinational therapy strategy using PIs and HDAC inhibitors, as opposed to sustained PI monotherapy, may be more effective in treating MLL leukemias by preventing the emergence of resistant cancer cells.

## Discussion

The relatively rapid acquisition of drug resistance to cancer therapies remains a crucial obstacle [36]. PIs have dramatically improved the treatment of multiple myeloma (MM) and other hematological malignancies, but relapses are frequent, and acquired resistance to treatment eventually emerges [37, 38]. Despite many mechanisms that have been proposed for PI resistance in MM, how PI resistance inevitably develops in MLL leukemia remains elusive. Here, we provide a unique mechanism underlying drug resistance in MLL leukemia and the efficacy of combination therapy. We show evidence that MLL is indispensable for latent tumor suppression-mediated vulnerability of MLL leukemic cells to PI treatment. Our study highlights the importance of preventing the emergence of treatment-induced drug-resistant cancer cells in cancer therapy. Conceivably, attenuating the acquisition of drug resistance would be an effective way to prevent treatment failure and relapse. To this end, combination and intermittent therapy strategies, as opposed to sustained monotherapy, should be utilized to prevent cancer cells from adapting a drug-resistant state [39, 40].

HDAC inhibitors have potential therapeutic effects on cancers and can be used in combination with a variety of chemotherapies and immunotherapies, and the synergy between bortezomib and HDAC inhibitors has been reported in MM and other hematological malignancy cells [41–43]. The drug interaction between bortezomib and HDAC inhibitors seems to be involving multiple mechanisms [44]. Nevertheless, our study suggests that preventing the emergence of resistant cancer cells may constitute a new rationale for this combinational therapy strategy. Histone acetylation induced by HDAC inhibitors can recruit wild-type MLL rather than MLL fusions on chromatin, thereby restoring MLL function and promoting cell cycle gene expression. These findings suggest that targeting MLL dysfunction by HDAC inhibition can be a promising strategy for treating the aggressive resistance in MLL leukemia. Of note, the combination of PI and HDAC inhibitor is being evaluated in a recent clinical trial of MLL-rearranged leukemia (NCT02553460), which demonstrated the promising efficacy of this combinational therapy strategy and the high clinical relevance of our study [20].

The discrepancy in p27 activation between acute and chronic PI treatment suggests that cell cycle deregulation in PI-resistant cells is achieved through an alternative mechanism. In our RNA-Seq data, chronic-induced dysregulation of many cell cycle genes, including cyclins, cyclin-dependent kinases (CDKs), and CDK inhibitors (CDKIs), which interact and influence each other in regulating cell cycle, were remarkably enriched and among the most significantly downregulated DEGs. This conflicting expression pattern of cell cycle genes suggesting that the cell cycle of PI-resistant MLL cells is in an inactive state and the expression level of p27 does not independently determine the cell cycle transition. Many observations suggest that p27 expression in MEFs and a few human cell lines is not completely associated with G1 arrest [45]. In addition, low levels of p27 observed in human tumors do not correlate with the proliferation index [46]. Our study demonstrated that the sensitivity of MLL leukemia cells to PI was related to the activation state of p27, and PI resistance was caused by a low replication state and the failure of p27 induction. Furthermore, we found that HDAC inhibitors not only induced the expression of p27 and other cell cycle genes, but also restored PI-induced p27 activation and thus restored cell sensitivity, suggesting that PIs selectively kill MLL cells during the cell cycle progression, and induced expression of p27 is necessary for the anti-MLL leukemia activity. Nevertheless, while HDAC inhibitors restored p27 induction in PI-resistant leukemic cells, they failed to repress leukemia development along in mice, indicating that additional pathways are required for killing leukemic cells by PI through MLL-fusion protein. Besides, we know very little about how these different cyclins, CDKs, and CDKIs differentially regulate the quiescence of MLL leukemic cells. To conclude, we are only beginning to appreciate the underlying importance of p27 and other cell cycle genes in the context of PI resistance. Thus, how such intrinsically and practically contradictory p27 protein dominates MLL cell sensitivity needs further exploration.

An expanding body of the literature emphasizes the contribution of epigenetic reprogramming in the development of inevitable drug resistance [47, 48]. We revealed that chronic PI treatment-induced epigenetic reprogramming caused unfavorable chromatin accessibility for MLL, resulting in less MLL to be recruited onto the chromatin. Subsequently, the loss of MLL function led to the dysregulation of cell cycle and the emergence of cell resistance. It is well recognized that cancer cells under drug stress primarily achieve cell fitness through modulating the signal transduction pathways governing these cellular stress response, and epigenetic changes are usually considered as secondary events [49]. Our study demonstrated that drug-induced epigenetic reprogramming might function as a driver in the adaptation process under therapy stress. Further elucidation of both epigenetic chromatin modification and accessibility may open new avenues for therapeutic targeting, including epigenetic modulators.

We and other groups have previously observed that MLL-r B-ALL cells, rather than AML cells, displayed selective sensitivity to PIs but not AML [18, 19]. Unlike ALL cells, the conflicting observations after the depletion of wild-type MLL allele in AML models have been the subject of intense research. Interestingly, while our results imply that MLL is the main mediator of the observed effects of bortezomib on cell cycle, which partially mediates the effect of H2Bub depletion in MLL-rearranged ALL cells, previous reports showed that the depletion of RNF20 but not MLL leads to inhibition of cell proliferation in MLL-rearranged AML cells, suggesting the effect of H2Bub depletion in AML cells is not mediated by MLL [13, 50]. This discrepancy indicated that the wild-type allele of MLL might play different roles in MLL-rearranged ALL and AML cells. Given the importance of discovering therapeutic targets in MLL-rearranged leukemia, further studies are needed to clarify these discrepancies.

In summary, we have demonstrated that MLL leukemic cells under PI treatment can generally and intrinsically acquire an MLL dysfunction-mediated cell cycle deregulation resistant state (Fig. 7h), which reveals the phenotype switching mechanism may extend beyond MLL-rearranged B-ALL leukemias. We also find that HDAC inhibitors can reverse PI resistance, which extends the therapeutic concept of MLL restoring. However, since MLL may play a different role in diverse cancer types, whether our proposed mechanism may extend to other cancers beyond MLL-rearranged leukemia remains an interesting topic requiring further investigation.

## Materials and methods

### Plasmid constructions

FLAG-tagged fragments consisting of MLL amino acids 1394–2665 (MLL^Inter^), 1394–1788 (PHD1–3-Bromo), 1394–1632 (PHD1–3), 1626–1987 (Bromo-PHD4), and 1626–1788 (Bromo) derived from wild-type MLL were inserted into eukaryotic expression vectors pCI-neo (Promega) for transient transfection assays. Transfection was performed according to the manufacturer’s protocol using FuGENE (Promega).

### Reagents

Bortezomib (Velcade), panobinostat (LBH589), and vorinostat (SAHA, MK0683) were obtained from Selleck Chemicals. The IRAK1/4 inhibitor was obtained from MedChemExpress. Cycloheximide was obtained from Sigma.

### Cell culture

Human pro-B MLL leukemia cell lines RS4;11 and SEM [51] were purchased from DSMZ. Cells were cultured in Gibco RPMI 1640 containing 10% FBS at 37 °C with 5% CO_2_ and were maintained between a density of 5 × 10^5^ cells/mL and 2 × 10^6^ cells/mL. SEM or RS4;11 cells were continuously treated with a sublethal dose of bortezomib (5 nM) for over 4 weeks. Cells were passaged and the inhibitor was replenished every 3 days. The half-maximal dosage effect (IC_50_) values were measured intermittently until the drug resistance was acquired. After the drug resistance development, the “Resistant” cells were collected for analysis.

### Cell viability and cell proliferation assays

The CellTiter 96 MTS assay (Promega) was used to determine the cytotoxicity of the relevant drugs and cell proliferation, according to the manufacturer’s instructions. Cell viability was measured with MTS assay 24 h after the addition of bortezomib with graded concentrations in triplicates. Cells were pretreated with IRAK1/4 inhibitor for 48 h before the measurement.

### Apoptosis and cell cycle assays

Apoptosis and cell cycle were measured using the PE Annexin V Apoptosis Detection Kit and APC BrdU Flow Kit from BD Pharmingen as described by the manufacturer, respectively. Cells staining with fluorochromes were acquired using flow cytometer and data were analyzed using FlowJo software.

### shRNA-mediated knockdown

Target sequences against human MLL C-terminus, ASH2L, RNF20, and a control scrambled sequence that has no significant homology with the human genome were inserted into the pLKO.1 vector, according to the manufacturer’s protocol (Addgene). Generated lentivirus carrying shRNA was used to infect target cells for 2 days, and the cells were subjected to puromycin selection at 2 μg/mL.

### RNA-Seq, ChIP, and qRT-PCR

Total RNA was extracted from Trizol according to the manufacturer’s instructions. The mRNA-seq library was sequenced using BGISEQ-500RS for 100-bp paired-end sequencing. After quality control, clean reads were aligned to the human genome (UCSC hg19) by Tophat2.1.0 with a maximum of two mismatches for each reads. Gene ontology analysis was performed using the KEGG database and DAVID (http://david.abcc.ncifcrf.gov) for pathway analysis. GSEA was executed using public software from the Broad Institute (http://software.broadinstitute.org/gsea). ChIP assays were performed following the described protocol [52]. Cellular RNA and precipitated DNA samples were reverse transcribed with random primers and detection was performed using 7500 Real-Time PCR Systems (Applied Biosystems). Primers used for qRT-PCR assays were listed in Table S7.

### Immunoblots

Human CD133-PE antibody was obtained from Miltenyi Biotec. Human CD45-FITC antibody was obtained from Biolegend. Mouse antibody against p27 was obtained from BD Biosciences. Rabbit antibodies against MLL^C180^ (D6G8N), MLL^N320^ (D2M7U), ASH2L, CD133, Ubiquityl-Histone H2B (Lys120) (H2Bub), Tri-Methyl-Histone H3 (Lys4) (H3K4me3), Histone H3, and mouse anti-Histone H2B antibody were purchased from Cell Signaling Technology. Rabbit anti-RNF20 antibody was purchased from ABclonal Technology. Rabbit anti-Acetyl-Histone H3 (Lys9/14) (H3K9/14ac) antibody was obtained from Thermo Fisher Scientific. Mouse anti-FLAG, β-Actin, and Tubulin antibodies were obtained from Sigma Aldrich. Antibodies were detected using the enhanced chemiluminescence method (PerkinElmer). Immunoblot signals were acquired with the Amersham Imager 600 (General Electric Company).

### Peptide pull-down and chromatin association assays

Peptide pull-down assays have been described previously [3]. In brief, individual biotin-conjugated histone H3 peptide (amino acids 1–21; 2 μg) or biotin-conjugated acetyl-histone H3 (Lys9/14) peptide (amino acids 1–21; 2 μg) (Millipore) were incubated with nuclear extracts, precipitated using streptavidin beads (GE Healthcare). The co-precipitated protein was detected by immunoblots. Chromatin-enriched fractions were purified as described previously [3].

### Mouse studies

NOD-SCID mice were purchased from Vital River Laboratory. MLL-r PDX were generated from two MLL-AF4-fusioned B-ALL patients. The characteristics of these MLL patients are detailed in Table S1. In total, 1 × 10^6^ bone marrow cells were collected and intravenously injected into NOD-SCID mice (6–8 weeks, male or female). Mice were then administered bortezomib intravenously at 1 mg/kg on a twice-weekly schedule, or administered vehicle, beginning 7 days after the xenograft. The percentage of leukemic cells in the peripheral blood of mice was monitored using CD45 antibody. To evaluate the in vivo efficacy of bortezomib combined with SAHA, 1 × 10^6^ SEM-resistant or MLL-r PDX-resistant cells were intravenously injected into NOD-SCID mice. Mice were then administered bortezomib intravenously at 1 mg/kg on a twice-weekly schedule, or administered SAHA intraperitoneally at 5 mg/kg three times per week, beginning 7 days after the xenograft. Mice undergoing bortezomib or SAHA monotherapy also received the vehicle. The percentage of leukemic cells in the peripheral blood of mice was monitored using CD133 and CD45 antibody, respectively [53]. Mice were sacrificed by inhalation of CO_2_ when they became moribund. For hematoxylin–eosin staining, the spleen and liver of mice were collected. Animal care and sacrifice were conducted according to methods approved by the Animal Care and Use Committee, the Center for Animal Experiments of Shanghai Jiao Tong University.

### Statistical analysis

The Student’s *t*-test was used to analyze the differences between the groups. Means were illustrated using a histogram with error bars representing ± the standard deviation (SD), and statistical relevance was evaluated using the following *P* values: *P < 0.05, **P < 0.01, or ***P < 0.001.

## Supplementary information

Supplementary Figures

Supplementary Tables

## Data Availability

All data generated or analyzed during this study are included in the paper and its supplementary information files. RNA-seq data are available in the Gene Expression Omnibus (GEO) database under accession number GSE138177.
