# Supplementary material for: Restoring MLL reactivates latent tumor suppression-mediated vulnerability to proteasome inhibitors
Source: Oncogene. 2020 Jul 30;39(36):5888–901. doi: 10.1038/s41388-020-01408-7 (PMC7471105; doi:10.1038/s41388-020-01408-7)
Supplement: Supplementary file 1 — Supplementary Figures [file 41388_2020_1408_MOESM1_ESM.docx]

**Supplementary Figures**


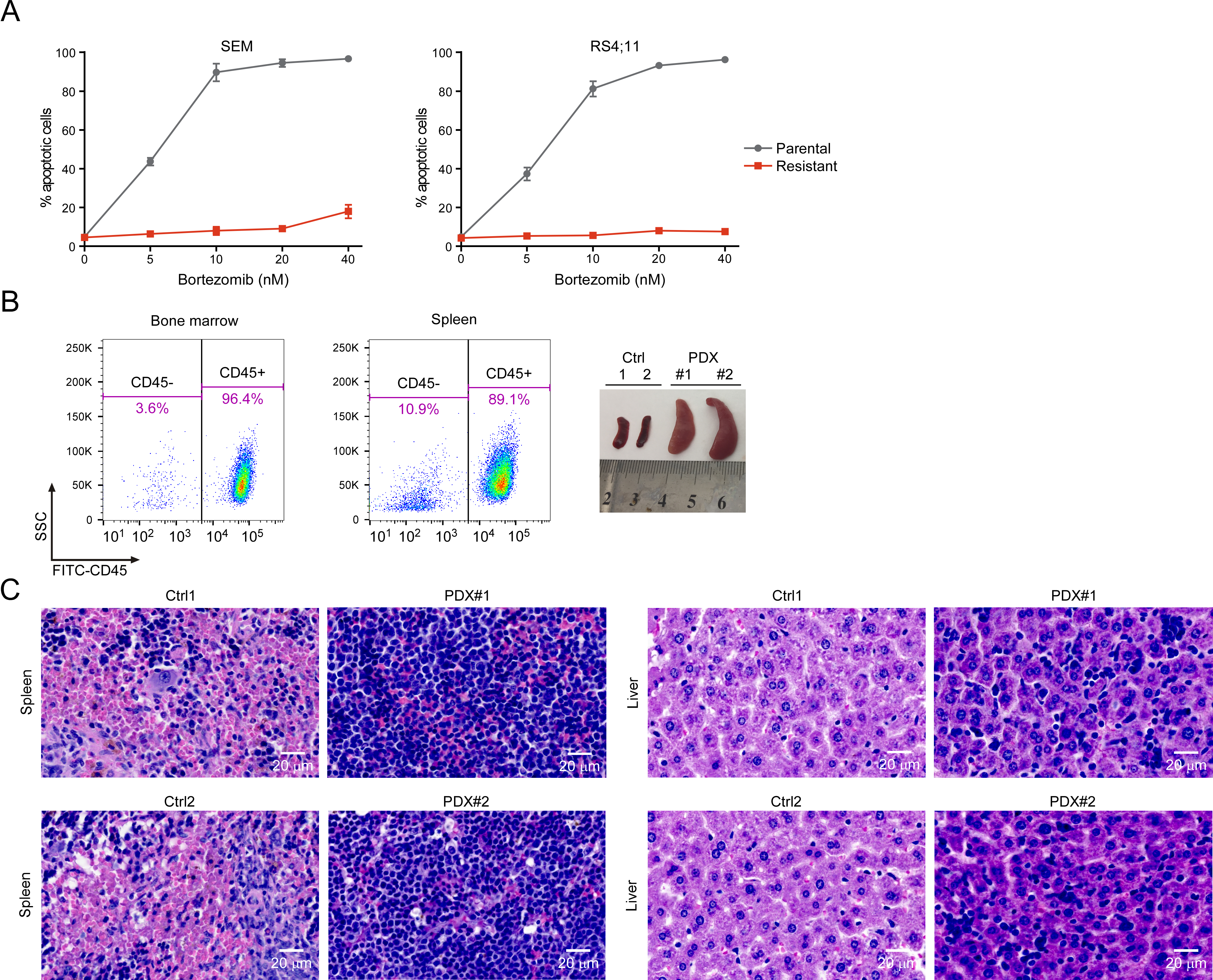


**Supplementary Figure 1. Characteristics of MLL-r cell lines and MLLr-PDX.** (**A**) The percentage of annexin V-positive cells was determined after a 16 hr treatment with bortezomib at the indicated concentrations. (**B**) Flow cytometric detection of CD45 expression of the bone marrow cells (left panel) and spleen cells (middle panel) from an MLL-arranged patient-derived xenograft (MLLr-PDX) mouse. The representative spleen of the control and PDX mice is shown (right panel). (**C**) Hematoxylin-eosin staining of the spleen and liver of the indicated control and PDX mice.

**
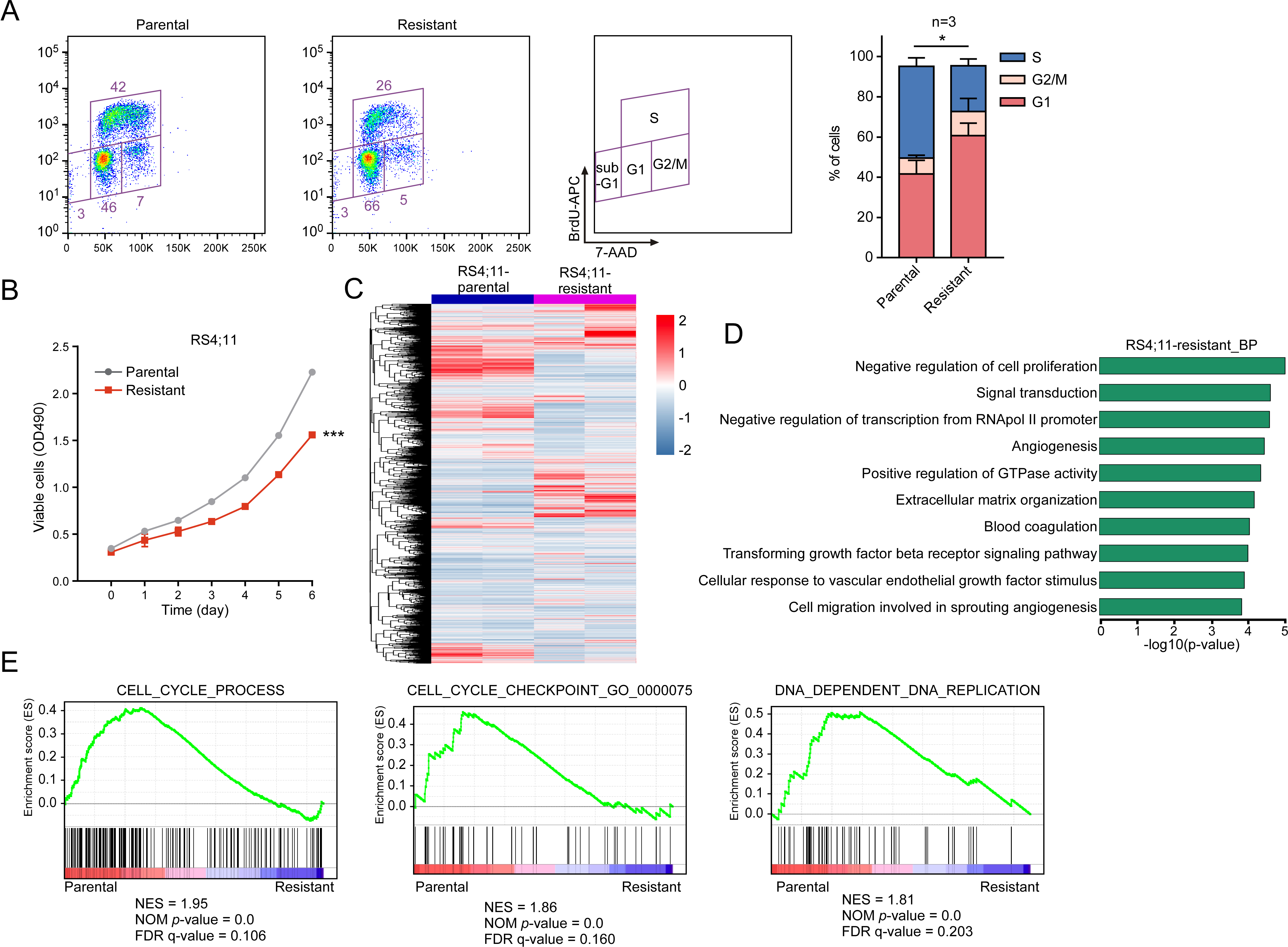
**

**Supplementary Figure 2. RNA sequencing analysis of parental and PI-resistant RS4;11 cells.** (**A**) Cell cycle profiling of RS4;11 parental and resistant cells. Stacked barplot shows the fraction of cells viable in G1, S and G2/M phases of the indicated cells (three independent biological replicates with three technical replicates each). (**B**) The proliferation of RS4;11 parental and resistant cells for 6 days. (**C**) Unsupervised hierarchical clustering heatmap of differentially expressed genes (DEGs) in SEM and RS4;11 parental and resistant cells, respectively. (**D**) Biological process (BP) enrichment analysis of DEG datasets obtained from RS4;11 parental and resistant cells. GO analysis was performed with DAVID, and items were ordered by *P*-value. (**E**) GSEA analysis of datasets obtained from RS4;11 parental and resistant cells. Normalized enrichment score (NES), nominal (NOM) *P*-value, and false discovery rate (FDR) are indicated. *, *P* < 0.05; ***, *P* < 0.001; two-tailed *t*-test. Data represent the means of triplicate reactions ± SD.

**
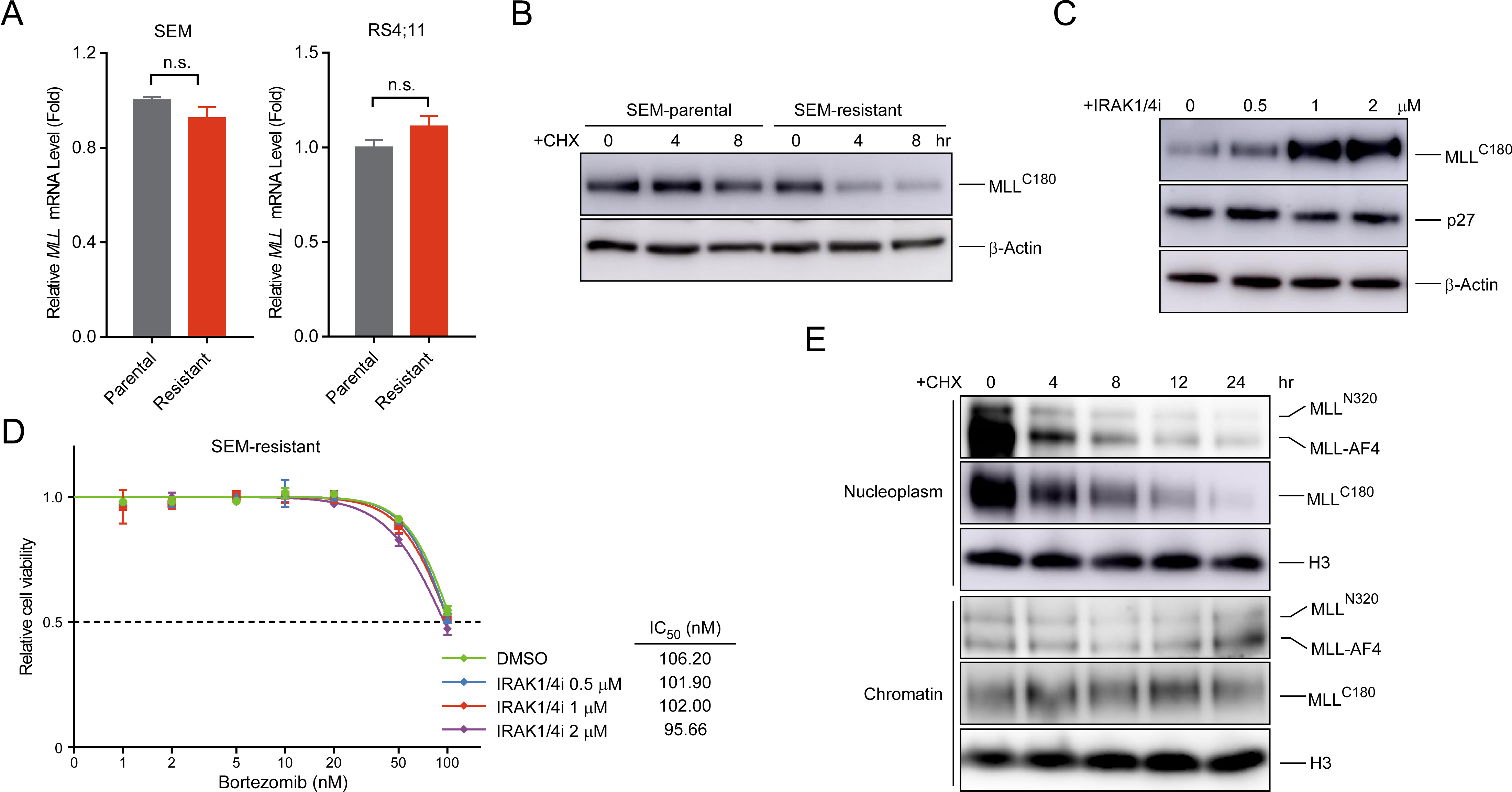
**

**Supplementary Figure 3. The unfavorable chromatin accumulation of MLL suppresses the effect of IRAK1/4 inhibitor on PI-resistant cells.** (**A**) *MLL* mRNA levels of the indicated cells. (**B**) SEM parental and resistant cells were treated with cycloheximide (CHX) for the indicated time. Immunoblots of the indicated antibodies were analyzed. (**C**) SEM-resistant cells treating with IRAK1/4 inhibitor for 48 hr and subjected to immunoblots. (**D**) SEM resistant cells treating with DMSO or IRAK1/4 inhibitor for 48 hr and cell viability was measured. The IC_50_ of different cells was quantified. **(E)** SEM cells were treated with CHX for the indicated time. Immunoblots of nucleoplasm and chromatin-bound fractions were analyzed. n.s., not significant; two-tailed *t*-test. Means of three PCRs ± SD are shown.


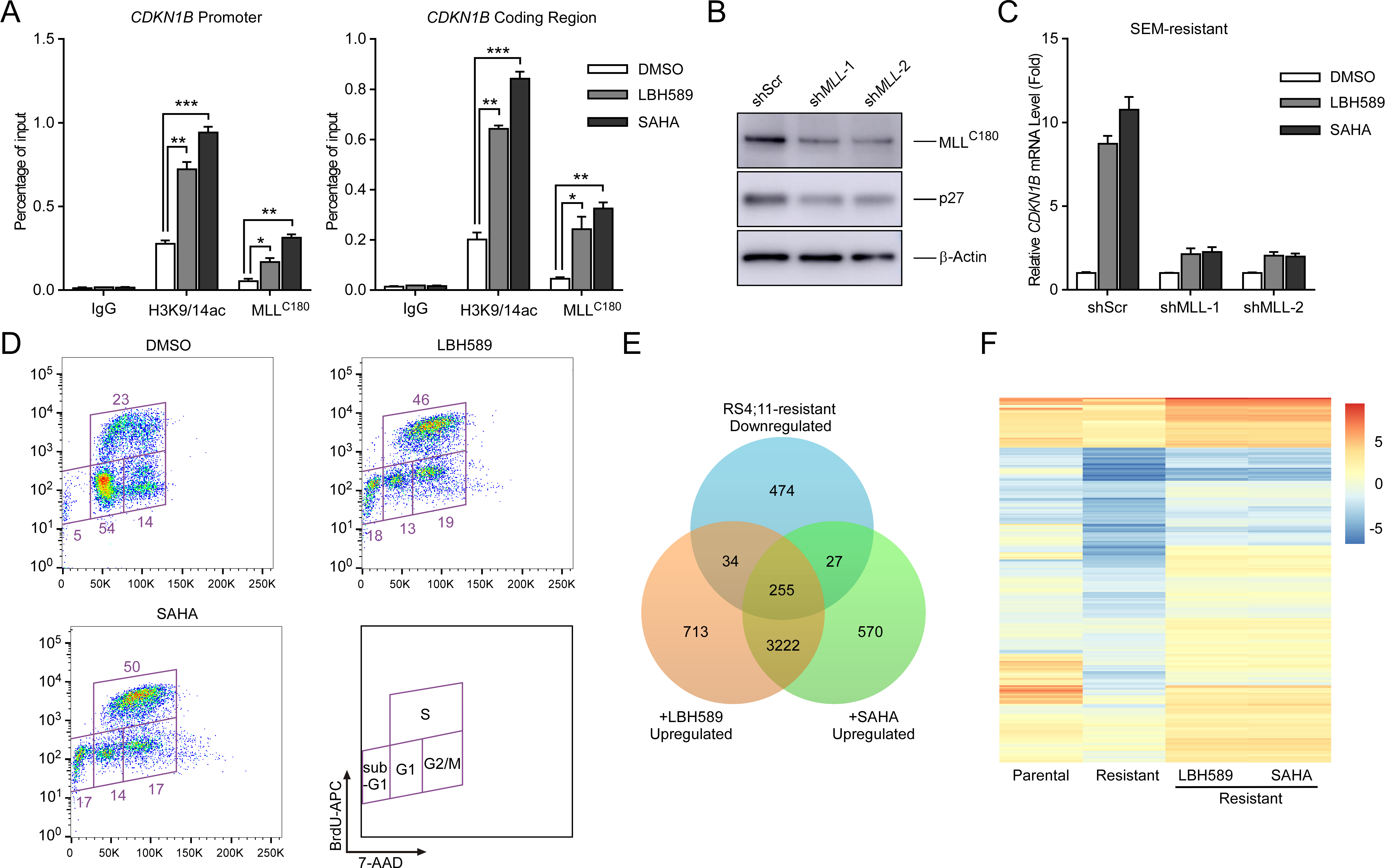


**Supplementary Figure 4. HDAC inhibitors restore cell cycle and gene expression patterns.** (**A**) ChIP analyses at the promoter and coding region of the *CDKN1B* locus in the indicated SEM-resistant cells treated with DMSO, LBH589 (5 nM), or SAHA (2 μM) for 20 hr. Assays were performed with the indicated antibodies and immunoprecipitates were subjected to quantitative PCR analyses. (**B**) The MLL and p27 protein levels of SEM-resistant cells infected by the indicated lentiviral vectors. (**C**) *CDKN1B* mRNA levels of the indicated cells treated with DMSO, LBH589 (5 nM), or SAHA (2 μM) for 20 hr. (**D**) Cell cycle profiles of SEM resistant cells treated with DMSO, LBH589 (5 nM), or SAHA (2 μM) for 20 hr. One representative of three independent experiments is shown. (**E, F**) Venn diagram (E) and heatmap of the overlapped 255 genes (F) of RS4;11 parental and resistant cells treating with DMSO, LBH589 (50 nM), or SAHA (2 μM) for 20 hr. *, *P* < 0.05; **, *P* < 0.01; ***, *P* < 0.001; two-tailed *t*-test. Data represent the means of triplicate reactions ± SD.


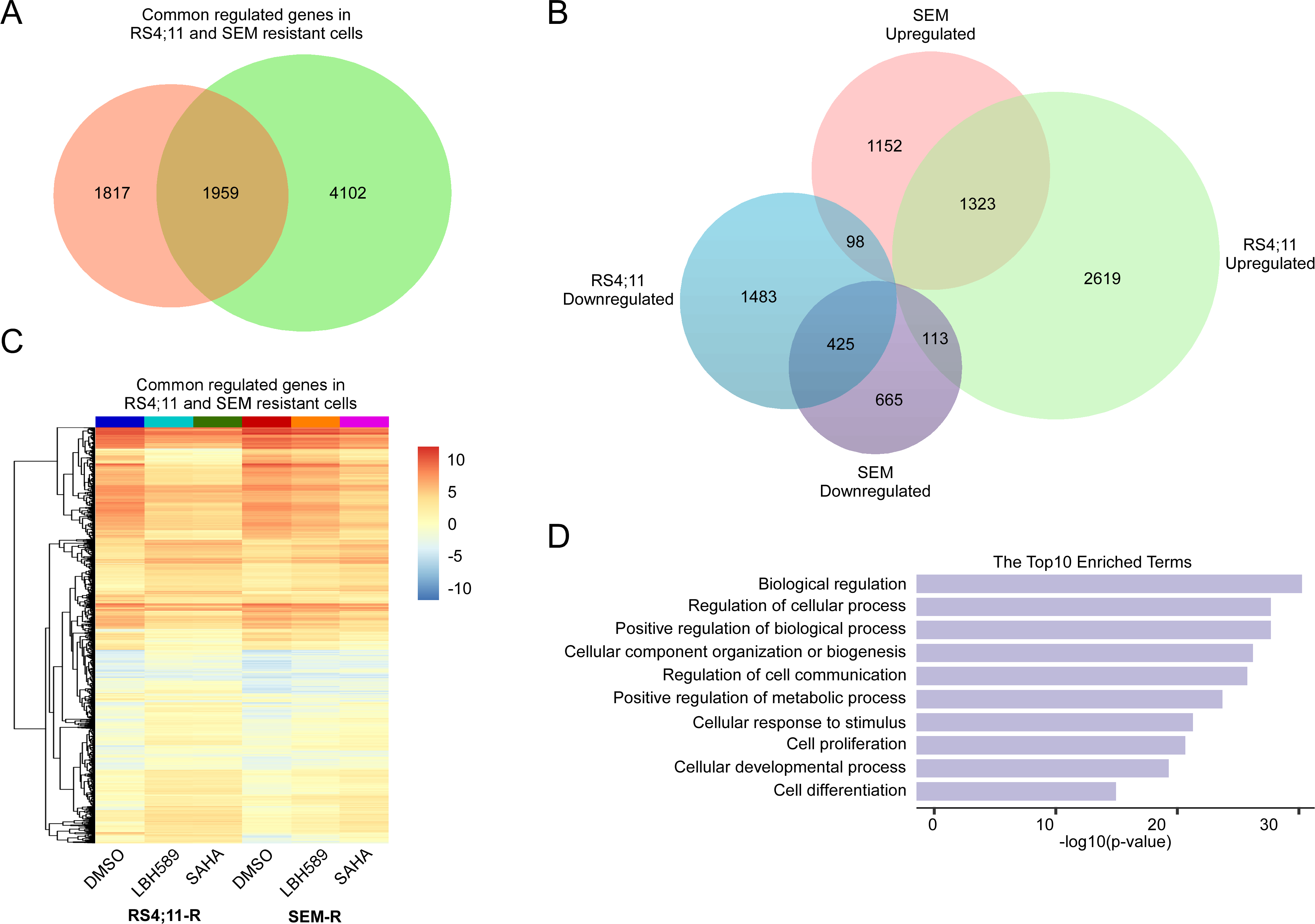


**Supplementary Figure 5. Overlap of genes between RS4:11 and SEM cells upon HDAC inhibitor treatment.** (**A**) Venn diagram analysis identifies 1959 common regulated genes by both HDAC inhibitors (LBH589 and SAHA) in SEM and RS4;11 resistant cells. (**B**) Venn diagram of deregulated genes in SEM and RS4;11 resistant cells by both HDAC inhibitors. 1323 genes were upregulated and 425 genes were downregulated in both resistant cells. (**C**) Unsupervised hierarchical clustering heatmap of 1959 common regulated genes by both HDAC inhibitors in SEM and RS4;11 resistant cells. (**D**) Biological process (BP) enrichment analysis of common regulated genes by both HDAC inhibitors in SEM and RS4;11 resistant cells. GO analysis was performed with DAVID, and items were ordered by *P*-value.


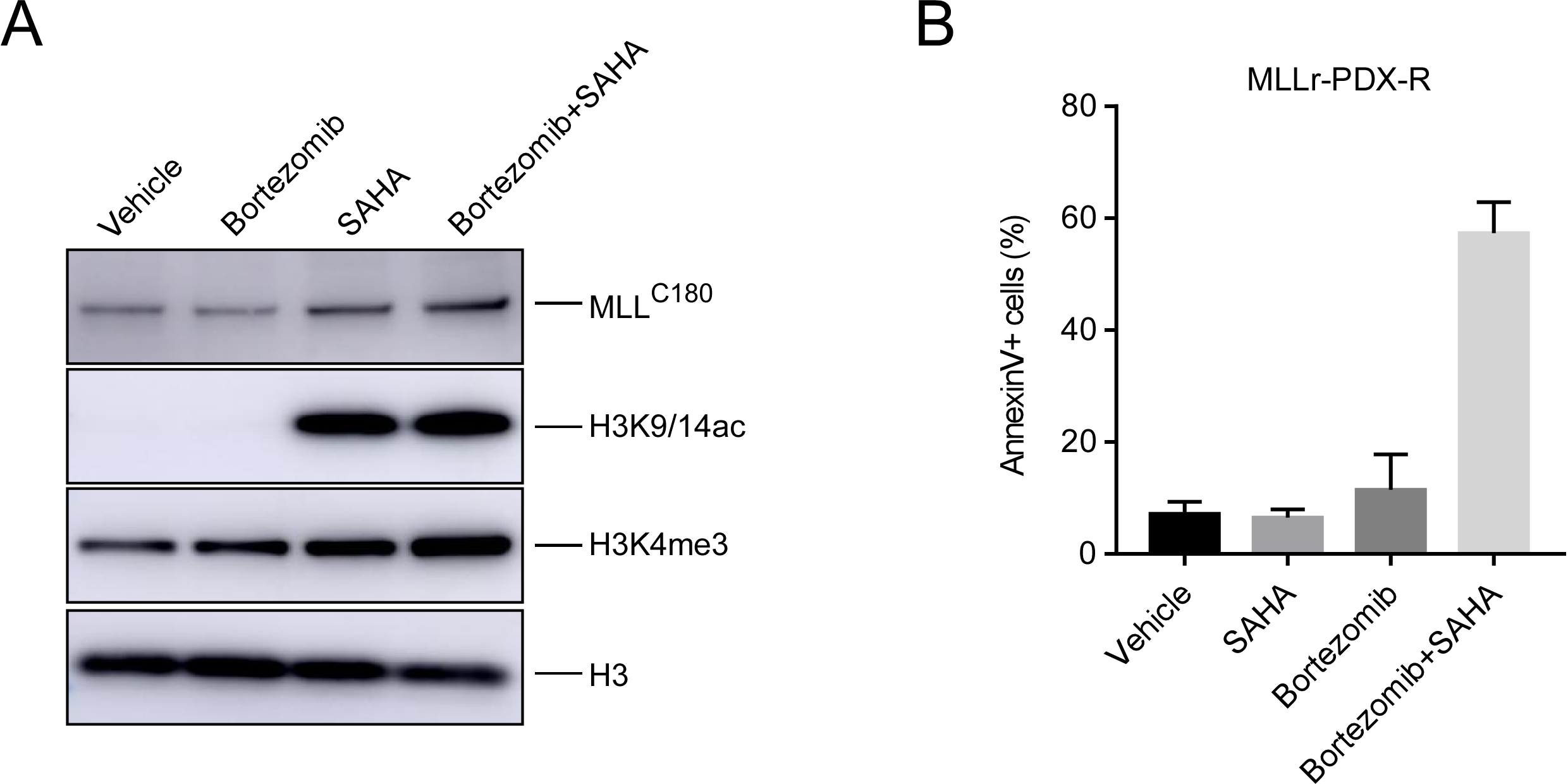


**Supplementary Figure 6. The combination of PI and HDAC inhibitor restores MLL expression and causes apoptosis.** (**A**) The xenograft mice transplanted with MLL-r PDX-resistant cells were treated with the indicated drugs. The bone marrow cells from xenograft mice were collected 30 days post transplant. Chromatin-bound fractions were purified and immunoblots of the indicated antibodies were analyzed. (**B**) Annexin V staining of the indicated PDX cells was assessed after a 12 hr treatment with 10 nM bortezomib.
